# Impact of an Educational Intervention on Nurses’ Knowledge of Port Catheter Management: A Quasi-Experimental Pre–Post Study

**DOI:** 10.3390/healthcare14142173

**Published:** 2026-07-18

**Authors:** Gamze Temiz, Nilay Ayberk, Elif Donmez, Tulay Ortabag, Burak Mete

**Affiliations:** 1 Hamidiye Faculty of Nursing, University of Health Sciences, Istanbul 34668, Turkey; ed.elifdonmez@gmail.com; 2 Kosuyolu Family Health Center, Istanbul 34718, Turkey; nilaymansur89@gmail.com; 3 Faculty of Health Sciences, İstanbul Topkapı University, Istanbul 34662, Turkey; tulayortabag@topkapi.edu.tr; 4 Department of Public Health, Faculty of Medicine, Cukurova University, Adana 01330, Turkey; bmete@cu.edu.tr

**Keywords:** implanted port catheter, nursing education, oncology nursing, knowledge, chemotherapy extravasation, patient safety, quasi-experimental study

## Abstract

**Highlights:**

**What are the main findings?**

- A structured educational intervention improved nurses’ short-term knowledge of implanted port catheter management.
- The intervention enhanced knowledge of key aspects of port catheter use, maintenance, and complication prevention.

**What are the implications of the main findings?**

- Structured educational interventions may support nurses’ knowledge of safe and evidence-based implanted port catheter management.
- Further controlled studies with longer follow-up are needed to determine whether knowledge gains are sustained and translate into improved clinical practice and patient outcomes.

**Abstract:**

**Background:** Implanted port catheters are widely used in oncology care to provide reliable long-term venous access. Safe management of these devices requires adequate nursing knowledge to prevent complications such as catheter dysfunction, infection, and chemotherapy extravasation. Despite their clinical importance, previous studies have demonstrated considerable deficiencies in nurses’ knowledge regarding implanted port catheter care. This study aimed to evaluate the short-term effect of a structured educational intervention on nurses’ knowledge of implanted port catheter management. **Methods:** A single-group pre–post quasi-experimental study was conducted in a public hospital in Istanbul, Türkiye, in July 2025. One hundred and one registered nurses completed the pre-test, and 73 participants who completed both assessments were included in the paired analysis. Participants attended a standardized evidence-based educational session covering port catheter insertion, maintenance, complication prevention, and extravasation management. Knowledge was assessed immediately before and after the intervention using a 20-item Port Catheter Knowledge Assessment Form. Data were analysed using the Wilcoxon signed-rank test and McNemar test. **Results:** The mean knowledge score increased significantly from 9.72 ± 6.14 before the intervention to 15.12 ± 1.97 after the intervention (*p* < 0.001), representing a large effect size (rank-biserial correlation = 0.883). Statistically significant improvements were observed in 15 of the 20 knowledge items following the educational intervention (*p* < 0.05). **Conclusions:** The educational intervention was associated with significant short-term improvements in nurses’ knowledge of implanted port catheter management. These findings support the value of structured educational programmes for improving knowledge; however, further studies are needed to determine whether these gains are sustained over time and translate into improvements in clinical practice and patient outcomes.

## 1. Introduction

Cancer remains a major global public health concern due to its high rates of morbidity and mortality [1]. Despite advances in treatment modalities, chemotherapy continues to be one of the most widely used therapeutic approaches in oncology care [2]. As the use of chemotherapy has increased, the need for reliable and long-term venous access has become essential [3]. To address this need, implanted port catheter systems have been developed as closed infusion devices designed for prolonged use. Implanted port catheter systems are subcutaneously implanted devices that provide direct access to central veins. They typically consist of a reservoir connected to a catheter and are commonly inserted via the subclavian or internal jugular veins [4,5,6].

Port catheters provide a safe and effective route for the administration of chemotherapy, blood products, and parenteral nutrition, as well as for blood sampling [4,7]. Their use offers several advantages, including reduced need for repeated venipuncture, lower patient discomfort, decreased anxiety, and improved quality of life. In addition, their closed system design contributes to reduced infection risk and allows patients to maintain daily activities [8,9].

Despite these advantages, the increasing use of port catheters has also led to a rise in associated complications [10]. Early complications include pneumothorax, hemothorax, arterial puncture, and cardiac arrhythmias, whereas late complications include infection, thrombosis, catheter dysfunction, fracture, and drug extravasation [4,10]. Among these, extravasation is one of the most serious complications, occurring when infused medications leak into surrounding tissues, potentially causing severe tissue damage. The reported incidence ranges from 0.1% to 6% in peripheral access and from 0.26% to 4.7% in central venous access [11,12,13,14,15]. Clinical manifestations include pain, swelling, erythema, and, in severe cases, tissue necrosis and long-term functional impairment. Such complications may compromise treatment continuity, increase healthcare costs, and negatively affect patients’ quality of life.

Nurses play a pivotal role in ensuring the safe use, maintenance, and monitoring of implanted port catheters. Their ability to recognize catheter-related complications, apply evidence-based maintenance protocols, and respond promptly to extravasation events is fundamental to patient safety. Nevertheless, previous studies have consistently reported important deficiencies in nurses’ knowledge regarding implanted port catheter care, including catheter maintenance procedures, flushing techniques, identification of complications, and appropriate management of chemotherapy extravasation [16,17,18,19,20,21,22]. These knowledge gaps may contribute to inconsistent clinical practice and increase the risk of preventable catheter-related complications.

Although several educational interventions have demonstrated positive effects on nurses’ knowledge of vascular access devices, the available evidence specifically addressing implanted port catheter management remains limited. Furthermore, relatively few studies have evaluated structured educational programmes focusing on both routine port catheter care and extravasation prevention using standardized knowledge assessments. Addressing these educational needs is essential for supporting evidence-based nursing practice and informing future educational strategies in oncology settings.

Therefore, this study aimed to evaluate the short-term effect of a structured educational intervention on nurses’ knowledge of implanted port catheter management using a pre–post quasi-experimental design.

## 2. Materials and Methods

### 2.1. Study Design and Setting

This study employed a single-group pre–post quasi-experimental design to evaluate the short-term effect of a structured educational intervention on nurses’ knowledge of implanted port catheter management. The study was conducted on 9 July 2025 in a public hospital in Istanbul, Türkiye.

Participants completed a knowledge assessment immediately before and immediately after the educational intervention to evaluate short-term changes in knowledge. Because the study was designed as a service-based educational evaluation, no control group was included. Therefore, the findings should be interpreted as evidence of an association between the intervention and knowledge improvement rather than definitive evidence of causality.

### 2.2. Participants and Eligibility Criteria

The study population consisted of registered nurses employed at the study hospital. Inclusion criteria were: (1) being a registered nurse; (2) being aged between 18 and 65 years; and (3) willingness to participate.

A total of 101 nurses completed the pre-test assessment. Of these, 73 participants who completed both pre- and post-test assessments were included in the final paired analysis. All eligible nurses present on the study day were invited to participate through face-to-face briefing.

Due to the service-based and pragmatic nature of the study, no formal a priori sample size calculation was performed; instead, all available and consenting nurses were included to maximise participation. Because questionnaires were completed anonymously, baseline characteristics of participants who did not complete the post-test could not be compared with those who completed both assessments.

### 2.3. Intervention (Educational Session)

The structured educational intervention was developed by the research team based on current evidence, national recommendations, and the relevant literature on implanted port catheter management and chemotherapy extravasation. The educational programme aimed to improve nurses’ knowledge of evidence-based port catheter care, including catheter insertion principles, maintenance procedures, flushing and locking techniques, nursing responsibilities, recognition of catheter-related complications, and the prevention and management of chemotherapy extravasation.

The educational session lasted approximately 60 min and was delivered by the principal investigator, who has experience in nursing education and implanted port catheter management. The session consisted of an evidence-based lecture supported by a standardized PowerPoint presentation, clinical photographs, current evidence-based recommendations, and case-based discussion. Participants were encouraged to ask questions throughout the session, allowing interactive discussion and clarification of clinical issues.

The educational content, teaching materials, and presentation format were standardized for all participants to ensure consistency of the intervention. No practical skills assessment or hands-on training was included as part of the educational programme.

### 2.4. Measures and Instruments

1. Socio-Demographic and Professional Characteristics Form: This form included variables such as age, gender, marital status, education level, years of professional experience, clinical unit, prior experience with port catheter procedures, previous training, and engagement with relevant literature.
2. Port Catheter Knowledge Assessment Form: A 20-item knowledge assessment form was developed by the researchers based on a comprehensive review of the current literature and evidence-based recommendations regarding implanted port catheter management and chemotherapy extravasation. The questionnaire was designed to assess nurses’ knowledge related to port catheter insertion, maintenance, nursing responsibilities, complication recognition, and extravasation management.

To establish content validity, the preliminary version of the questionnaire was reviewed by five faculty members with expertise in oncology nursing, nursing education, and clinical research. The questionnaire was revised in accordance with their recommendations to improve the clarity, relevance, and comprehensiveness of the items.

Each item was scored as 1 for a correct response and 0 for an incorrect response or “I do not know”, resulting in a total score ranging from 0 to 20. Items 3, 6, 8, and 11 were reverse-coded. Higher scores indicated greater knowledge of implanted port catheter management.

Because the instrument was developed as a criterion-referenced knowledge test rather than a psychometric scale intended to measure a latent construct, internal consistency reliability (e.g., Cronbach’s alpha) was not assessed. Instead, emphasis was placed on content validity through literature review and expert evaluation.

### 2.5. Data Collection and Management

Participants completed the pre-test immediately before and the post-test immediately after the educational session. Data were collected anonymously and linked using unique numeric codes to allow paired comparisons.

Data entry was performed independently by two researchers and cross-checked for accuracy. Missing responses were treated as incorrect for total score calculation, and pairwise available data were used for item-level analyses.

### 2.6. Statistical Analysis

Data were analysed using IBM SPSS Statistics version 26.0 (IBM Corp., Armonk, NY, USA). Descriptive statistics (frequency, percentage, mean, standard deviation, median, interquartile range, minimum, and maximum) were used to summarise participant characteristics and knowledge scores.

The normality of data distribution was assessed using the Shapiro–Wilk test, along with visual inspection of histograms and Q–Q plots. As the data were not normally distributed, non-parametric tests were applied.

The Wilcoxon signed-rank test was used to compare pre- and post-intervention total knowledge scores. Effect size was calculated using the rank-biserial correlation coefficient and interpreted according to standard thresholds. Differences in correct response rates for individual items were analysed using the McNemar test.

**Figure 1:**
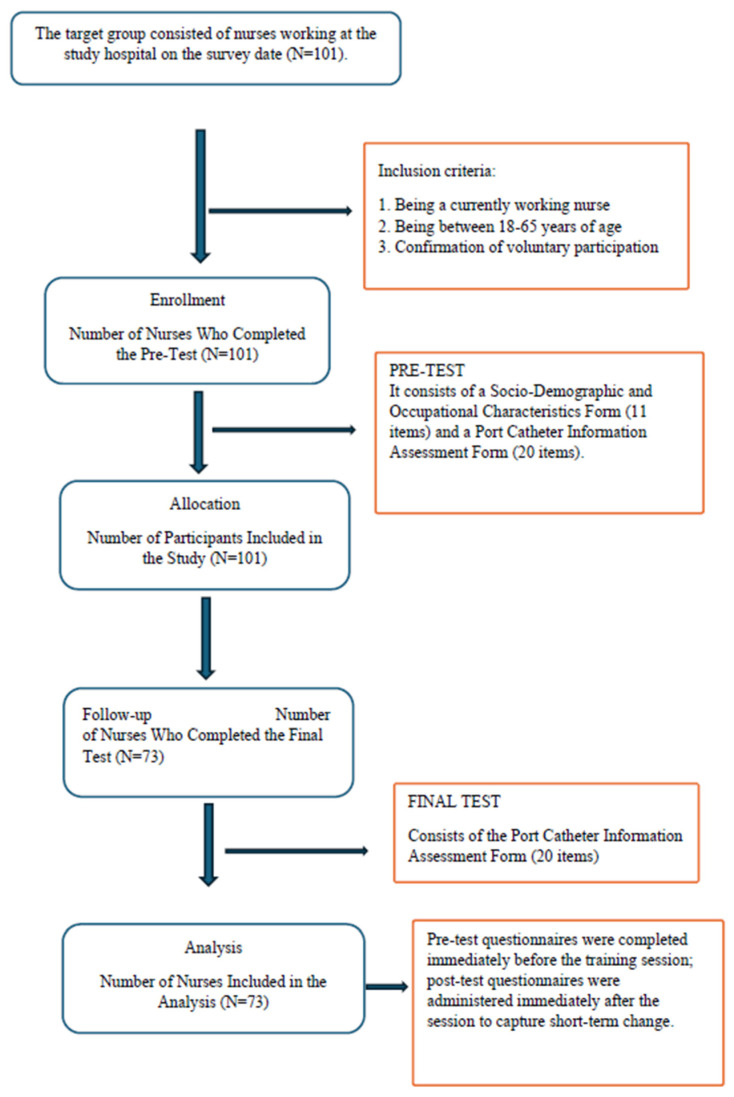
Flow diagram of participants.

A two-tailed *p*-value < 0.05 was considered statistically significant. Participant flow is shown in Figure 1.

### 2.7. Ethical Considerations

Ethical approval was obtained from the University of Health Sciences Hamidiye Scientific Research Ethics Committee (Approval No: 25/495; Approval Date: 29 May 2025). Written informed consent was obtained from all participants prior to data collection. The study was conducted in accordance with the principles of the Declaration of Helsinki. Participation was voluntary, and all data were collected anonymously without personal identifiers.

## 3. Results

The mean age of the participants was 25.26 ± 1.55 years. The participants had a mean professional experience of 1.00 ± 0.53 years and a mean oncology experience of 5.00 ± 1.48 months. On average, they reported managing 10.00 ± 2.73 patients with implanted port catheters per day. The majority were female (83.2%), held a bachelor’s degree (89.1%), were actively employed (71.3%), and reported following current scientific publications (67.3%) (Table 1).

More than half of the participants (58.4%) had not received prior training on port catheter care. In addition, 38.6% were unaware of nurses’ responsibilities in port catheter management, and 37.7% reported frequent or very frequent exposure to patients with port catheters in their clinical practice (Table 2).

A statistically significant improvement in knowledge scores was observed following the educational intervention. The mean pre-intervention score was 9.72 ± 6.14, increasing to 15.12 ± 1.97 post-intervention. Similarly, the median score increased from 12.5 to 15.0. The interquartile range decreased from 11.25 to 2.00, indicating reduced variability and a more homogeneous distribution of knowledge scores after the intervention. The minimum score increased from 0 to 7, while the maximum score increased from 18 to 20, reflecting overall improvement across participants.

The Wilcoxon signed-rank test confirmed that the difference between pre- and post-intervention scores was statistically significant (*p* < 0.001), with a large effect size (rank-biserial correlation = 0.883). These findings indicate a strong positive impact of the educational intervention on nurses’ knowledge levels (Table 3).

At the item level, a statistically significant improvement was observed in 15 of the 20 knowledge questions (*p* < 0.05) following the intervention (Table 4, Figure 1).

## 4. Discussion

This study demonstrated that a structured educational intervention was associated with significant short-term improvements in nurses’ knowledge of implanted port catheter management. Participants achieved significantly higher post-test knowledge scores immediately after the educational session, indicating that the intervention was effective in improving knowledge acquisition in the short term. These findings are consistent with previous studies demonstrating the positive impact of structured educational programmes on nurses’ knowledge of vascular access management and complication prevention. However, because knowledge was assessed immediately after the intervention, the present findings should be interpreted as evidence of short-term knowledge gain rather than long-term knowledge retention or changes in clinical performance.

A substantial proportion of participants (58.4%) reported not having received prior training on port catheter care. This finding is consistent with previous studies highlighting gaps in nursing education regarding vascular access management. For instance, Atay et al. (2023) reported that 74.5% of nurses had not received in-service training on extravasation management [20]. Similarly, Hussain et al. (2025) found that nurses demonstrated limited baseline knowledge of port catheter structure and function prior to training [21], while Galal et al. (2024) reported low levels of knowledge regarding port catheter indications, maintenance, and care before implementation of evidence-based guidelines [22]. Collectively, these findings underscore the presence of persistent educational deficiencies and highlight the need for standardized training programmes in this field.

The large effect size observed between pre- and post-intervention scores in the present study indicates a strong impact of the educational programme on knowledge acquisition. This finding is consistent with previous intervention studies. Mousavi et al. (2024) demonstrated that structured educational interventions significantly improved nurses’ knowledge and practices regarding peripheral intravenous catheter management [23]. Similarly, Chan et al. (2020) reported sustained improvements in nurses’ knowledge and guideline adherence six months after implementation of an evidence-based extravasation prevention programme [24]. These studies support the effectiveness of structured and guideline-based education in improving vascular access-related competencies. Although improved knowledge is an essential prerequisite for evidence-based nursing practice, this study did not evaluate whether these gains translated into behavioural change, procedural competence, adherence to clinical guidelines, or improvements in patient outcomes. Future longitudinal studies incorporating objective assessments of clinical performance are therefore warranted.

Although most knowledge items showed statistically significant improvement following the educational intervention, several items did not demonstrate significant change. These findings may indicate that some concepts were already familiar to participants before the intervention or that these topics require alternative educational strategies and repeated reinforcement to achieve measurable improvement. Future studies should investigate these knowledge domains in greater detail.

Item-level analysis revealed significant improvements in 15 of 20 knowledge items, suggesting that the intervention enhanced both general and specific domain knowledge. This is particularly important, as competence in port catheter management requires not only theoretical understanding but also the ability to apply knowledge in clinical decision-making. Comparable findings have been reported in studies focusing on catheter-related infection control and urinary catheter care, where structured education significantly improved both knowledge and attitudes among nurses [25,26,27]. The relatively young age and limited professional experience of the participants should also be considered when interpreting the findings. Although participants reported regular exposure to patients with implanted port catheters in their daily clinical practice, their average professional experience was one year and their mean oncology experience was five months. These characteristics may partly explain the relatively low baseline knowledge scores observed before the educational intervention and further support the need for structured educational programmes, particularly for early-career nurses.

Notably, post-intervention improvements were observed in critical areas such as the recognition of early complications following port catheter insertion and appropriate clinical responses to extravasation events. Given that extravasation can lead to severe tissue damage, necrosis, and long-term functional impairment if not promptly identified and managed, enhancing nurses’ knowledge in this area is directly linked to improved patient safety and treatment outcomes [3,4,9]. Therefore, strengthening educational interventions in this domain may contribute to improving nurses’ knowledge and could support safer evidence-based practice. However, additional studies are needed to determine whether improved knowledge results in measurable reductions in catheter-related complications.

The integration of national and institutional guidelines into nursing education may further support nurses’ knowledge and encourage evidence-based clinical decision-making. Future studies should evaluate whether such educational strategies are associated with improved adherence to clinical guidelines and better patient-related outcomes [11]. Such approaches may be particularly valuable in high-risk clinical environments such as oncology units.

Despite these positive findings, the short-term nature of the evaluation limits conclusions regarding long-term knowledge retention and sustained behavioural change. Future research should investigate the durability of educational effects over time and evaluate whether improved knowledge translates into measurable improvements in clinical practice and patient outcomes. Additionally, multicentre studies and objective assessments of clinical skills would enhance the generalisability and robustness of findings.

Finally, structured educational interventions should be integrated into continuous professional development programmes for nurses. As highlighted in the literature, ongoing education plays an important role in supporting nurses’ knowledge and promoting evidence-based clinical decision-making. Future studies should determine whether improvements in knowledge are sustained over time and translate into enhanced clinical practice, adherence to evidence-based guidelines, and improved patient outcomes [25,26,27].

### Strengths and Limitations

This study has several strengths. It is among the few studies to evaluate nurses’ knowledge of implanted port catheter management in a real clinical setting using a structured educational intervention. The pre–post design enabled direct within-participant comparisons of short-term knowledge change, and the use of a standardized 20-item knowledge assessment tool enhanced methodological consistency.

However, several limitations should be acknowledged. The sample primarily consisted of young, female, and university-educated nurses, which may limit the generalizability of the findings to more diverse nursing populations. In addition, the study evaluated only short-term knowledge acquisition and did not assess long-term knowledge retention. The absence of a control group limits causal inference, as improvements may have been influenced by testing effects or short-term recall.

Furthermore, approximately 28% of participants did not complete the post-test assessment, which may have introduced attrition bias. Because questionnaires were completed anonymously, baseline characteristics of completers and non-completers could not be compared.

Finally, the study assessed knowledge only and did not include objective measures of clinical competence, procedural performance, adherence to clinical guidelines, or patient outcomes. The single-centre design may further limit the external validity and generalizability of the findings.

## 5. Conclusions

The structured educational intervention significantly improved nurses’ knowledge regarding implanted port catheter management and extravasation prevention. These findings highlight the importance of regular evidence-based educational programmes in strengthening nursing competence and promoting patient safety in oncology care. Future studies should evaluate long-term knowledge retention and the impact of such interventions on clinical practice outcomes.

## Figures and Tables

**Table 1:** Distribution of sociodemographic variables (*n* = 101).

Variables	*n*	%
Gender		
Male	17	16.8
Female	84	83.2
Education Level		
Bachelor’s Degree	91	89.1
Master’s Degree	10	9.9
Employment Status		
Not currently employed in clinical practice	29	28.7
Currently employed in clinical practice	72	71.3
Following Current Publications		
Yes	68	67.3
No	33	32.7
Variable	Mean ± SD	
Age (years)	25.26 ± 1.554	
Professional experience (years	1.0 ± 0.530	
Oncology experience (months)	5.0 ± 1.477	
Average number of patients with implanted port catheters managed per day	10.0 ± 2.732	

**Table 2:** Status of receiving education on port catheters (*n* = 101).

Variables	*n*	%
Received prior education on port catheters		
Yes	42	41.6
No	59	58.4
Source of Education (*n* = 42)		
During formal coursework	15	14.9
In-service training programs	15	14.9
Self-researched	4	4.0
Course/Congress	5	5.0
Internship	5	5.0
Current Unit of Employment		
Other	81	81.2
Oncology/Hematology Units	20	19.8
Do you know the nurse’s responsibilities regarding port catheters?		
Yes	62	61.4
No	39	38.6
Frequency of encountering patients with port catheters during your career		
Very frequently	15	14.9
Frequently	23	22.8
Rarely	46	45.5
Never	17	16.8
Would you like updated information on port catheters to be included in in-service training programs?		
Yes	95	94.1
No	6	5.9

**Table 3:** Comparison of pretest and post-test correct responses.

	Mean	Median	SD	IQR	Min	Max	*p*	Rank Biserial Correlation
Pretest score	9.72	9.5	6.14	11.25	0.00	18.0	<0.001	0.883
Post-test score	15.12	15.0	1.97	2.00	7.00	20.0		

Wilcoxon test.

**Table 4:** Comparison of pretest and post-test item responses.

Item	Pre-Test Correct *n* (%) (*n* = 101)	Post-Test Correct *n* (%) (*n* = 73)	Change (%)	*p*-Value
S1	71 (70.3%)	67 (95.7%)	25.4	<0.001
S2	44 (43.6%)	56 (78.9%)	35.3	<0.001
S3	7 (6.9%)	11 (15.5%)	8.6	0.227
S4	66 (65.3%)	67 (94.4%)	29.1	<0.001
S5	73 (72.3%)	69 (97.2%)	24.9	<0.001
S6	13 (12.9%)	16 (22.5%)	9.6	0.180
S7	58 (57.4%)	64 (90.1%)	32.7	<0.001
S8	22 (21.8%)	21 (29.6%)	7.8	0.064
S9	64 (63.4%)	65 (91.5%)	28.1	<0.001
S10	40 (39.6%)	63 (88.7%)	49.1	0.864
S11	24 (23.8%)	30 (42.3%)	18.5	0.011
S12	54 (53.5%)	45 (63.4%)	9.9	0.265
S13	60 (59.4%)	68 (95.8%)	36.4	<0.001
S14	49 (48.5%)	60 (84.5%)	36.0	<0.001
S15	41 (40.6%)	56 (78.9%)	38.3	<0.001
S16	59 (58.4%)	66 (94.3%)	35.9	<0.001
S17	60 (59.4%)	69 (98.6%)	39.2	<0.001
S18	36 (35.6%)	38 (53.5%)	17.9	0.041
S19	58 (57.4%)	68 (67.3%)	9.9	<0.001
S20	63 (62.4%)	69 (68.3%)	5.9	<0.001

McNemar test. Note: Post-test participation was lower than pretest participation; analyses and percentages are based on valid percentages.

## Data Availability

The datasets generated and/or analysed during the current study are available from the corresponding author upon reasonable request. Access to the data is subject to institutional policies and the protection of participant confidentiality.
